# Short sleep duration, complaints of vital exhaustion and perceived stress are prevalent among pregnant women with mood and anxiety disorders

**DOI:** 10.1186/1471-2393-12-104

**Published:** 2012-10-03

**Authors:** Chunfang Qiu, Bizu Gelaye, Neway Fida, Michelle A Williams

**Affiliations:** 1 Center for Perinatal Studies, Swedish Medical Center, 1124 Columbia Street, Suite 750, Seattle, Washington, USA; 2 Department of Epidemiology, Harvard School of Public Health, Boston, Massachusetts, USA; 3 Department of Epidemiology, School of Public Health, University of Washington, Seattle, Washington, USA

## Abstract

**Background:**

Psychiatric disorders have been associated with sleep disorders in men and non-pregnant women, but little is known about sleep complaints and disorders among pregnant women with psychiatric disorders.

**Methods:**

A cohort of 1,332 women was interviewed during early pregnancy. We ascertained psychiatric diagnosis status and collect information about sleep duration, daytime sleepiness, vital exhaustion and perceived stress. Logistic regression procedures were used to estimate odds ratios (ORs) and 95% confidence intervals (CIs).

**Results:**

Approximately 5.1% of the cohort (n=68) reported having a physician-diagnosis of mood or anxiety disorder before interview. Compared with women without a psychiatric diagnosis, the multivariable-adjusted OR (95% CI) for short sleep duration in early pregnancy (≤6 hours) were 1.95 (1.03-3.69). The corresponding OR (95%CI) for long sleep duration (≥9 hours) during early pregnancy was 1.13 (0.63-2.03). Women with psychiatric disorders had an increased risk of vital exhaustion (OR=2.41; 95%CI 1.46-4.00) and elevated perceived stress (OR=3.33; 95%CI 1.89-5.88). Observed associations were more pronounced among overweight/obese women.

**Conclusions:**

Women with a psychiatric disorder were more likely to report short sleep durations, vital exhaustion and elevated perceived stress. Prospective studies are needed to more thoroughly explore factors that mediate the apparent mood/anxiety-sleep comorbidity among pregnant women.

## Background

Sleep disturbance, a significant health issue among pregnant women, is associated with several adverse pregnancy outcomes including prolonged labor, increased cesarean section rates, preeclampsia, preterm birth and postpartum depression [1-6]. Available studies suggest that up to 25% of pregnant women report significant sleep disturbances in the first trimester, with rates climbing to nearly 75% by the third trimester [7]. Pregnancy associated physiological and hormonal changes are known to contribute to increased prevalence and severity of sleep disturbance among pregnant women [7-10].

A substantial literature indicates that sleep disturbances and psychiatric disorders (e.g., mood and anxiety disorders) are closely related, with psychiatric disorders having considerable impacts on sleep quality [11-17]. Sleep disturbance is both a defining feature of depression diagnosis [7], and is a prodromal symptom of both new and recurrent depressive episodes [18,19]. Mood and anxiety disorders are known to be prevalent among reproductive age and pregnant women with a figure of 12.4% (range: 10% - 25%) [20-22]. However, despite these observations, the information concerning the association of mood and anxiety disorders with sleep characteristics during pregnancy is relatively limited. We are aware of several studies that have assessed maternal early pregnancy sleep characteristics among depressed pregnant women [19,23-28]. However, many of these studies did not account for potential confounding factors. To extend the literature, we assessed the relative risks of short and long sleep duration, as well as excessive daytime sleepiness, vital exhaustion and elevated perceived stress during early pregnancy among women with and without a history of physician diagnosed mood and anxiety disorders. We hypothesized that pregnant women with a history of physician diagnosed clinical mood or anxiety disorders were more likely than women with no such history to report shorter sleep durations, and to report higher frequencies of excessive daytime sleepiness, vital exhaustion and perceived stress.

## Methods

### Study Population and Setting

This analysis is based on data collected from a cohort of women attending prenatal care clinics affiliated with Swedish Medical Center in Seattle, Washington, USA. Eligible women started prenatal care before 20 weeks gestation, were 18 years of age or older, could speak and read English, and planned to deliver at the study hospital. Participants reported socio-demographic, behavioral, and health characteristics in a structured interview completed between 8–19 weeks (mean and standard deviation: 16.0±2.6) weeks gestation. After delivery, study personnel abstracted data from participants’ hospital labor and delivery medical records and clinic records. Between December 2003 and July 2006, 1,393 (82%) of 1,685 approached women consented to participate. We sequentially excluded 12 women with early pregnancy losses prior to the interview and 49 women who did not complete the interview. A total of 1,332 women remained for analysis. All study procedures were approved by the Institutional Review Board of Swedish Medical Center. All participants provided written informed consent.

### Data Collection

Interviewer-administered questionnaires were completed by participants in the analytical population at a mean gestational age of 16 weeks. Characteristics measured using the questionnaire included maternal age, height, pre-pregnancy weight, reproductive and medical history including her history of psychiatric disorders, average nightly sleep duration during pregnancy, vital exhaustion during early pregnancy, and perceived stress during early pregnancy. Information pertaining to participants’ psychiatric diagnoses was derived from self-reported medical histories with verification of diagnoses in medical records. Medical records included information about type of psychiatric disorder (e.g., depression, bipolar disorder, or anxiety disorders) and the timing of diagnosis. A total of 68 women were identified as having a diagnosis of a psychiatric disorder in this cohort. Sixty of these women had the diagnosis prior to the index pregnancy; the remaining 8 women had the diagnosis made during the first 20 weeks of the index pregnancy. No participant reported having bi-polar disorder. Fifty-seven of the sixty women with psychiatric disorders diagnosed before pregnancy reported using psychotropic medications during pregnancy. Seven of the 8 women with psychiatric diagnoses made in early pregnancy reported taking psychotropic medications in early pregnancy.

Maternal average nightly sleep duration during early pregnancy was ascertained by asking women the following question: “Since becoming pregnant, how many hours per night do you sleep?” Responses were reported as integers. We classified participants as short (≤ 6hours); normal (7–8 hours); and long (≥ 9 hours) duration sleepers. These categorizations were decided upon *a priori*, as decisions were guided by cut-points used by previously investigators, particularly those who focused on sleep problems among pregnant women [29].

Maternal report of vital exhaustion in early pregnancy was ascertained by asking participants the following question: “Since becoming pregnant, how often did you experience a sense of exhaustion (except after exercise)?” Response choices were: (1) never; (2) somewhat infrequently (about once monthly); (3) frequent (2–3 times per month); and (4) very frequent (almost weekly). For multivariable analyses, we collapsed responses into a dichotomous variable with “no” comprising the responses never and somewhat infrequently, and “yes” comprising the responses frequent and very frequent.

We used the Epworth Sleepiness Scale (ESS) [30] to assess maternal daytime sleepiness status during early pregnancy. Participants were asked to rate, on a scale of 0 to 3 (where 0=would never doze; 1=slight chance of dozing; 2=moderate chance of dozing; and 3=high chance of dozing), the likelihood they would fall asleep or doze off in each of the following eight common situations: sitting and reading; watching television; sitting, inactive in a public place (e.g., a theater or meeting); as a passenger in a car for an hour without a break; lying down to rest in the afternoon when circumstances permit; sitting and talking to someone; sitting quietly after lunch without alcohol; and in a car, while stopped for a few minutes in traffic. The scale yields a total score that ranges from 0 to 24, with higher ESS scores representing more severe subjective daytime sleepiness, respectively. Scores of 0–9, 10–12 and 13–24 are considered to represent normal, borderline and abnormal daytime sleepiness. For multivariable analyses, we created a dichotomous variable where participants with scores of ≥13 were classified as having excessive daytime sleepiness.

We used a modified abbreviated version of the Perceived Stress Scale (PSS)*,* to measure the subjective experiences of stress and coping with stress using the past three month as timeframe. This timeframe corresponds to the time period since becoming pregnant (i.e., the first trimester). The abbreviated version, an economical and simple psychological instrument to administer, measures the degree to which situations in participants’ life over the period of observation are appraised as stressful [31]. Items were selected to detect how unpredictable, uncontrollable, and overloaded participants find their lives. For multivariate analyses, we classified participants with scores ≥7 as having elevated perceived stress; those participants with scores <7 served as the reference group.

### Statistical Analysis

We compared frequency distributions of socio-demographic, lifestyle, behavioral and medical history characteristics of participants according to her psychiatric diagnosis status. We used unadjusted and multivariable-adjusted logistic regression models to calculate odds ratios (ORs) and 95% confidence intervals (CIs) of the association between psychiatric disorder and sleep or stress variables. Separate models were fitted for each sleep complaint or stress variable. In multivariable models, we adjusted for maternal age (continuous), parity (nulliparous, multiparous), history of pre-gestational hypertension (no, yes), and pre-pregnancy body mass index (≤18.5, 18.5-24.9, 25–29.9, ≥30 kg/m^2^). Additional adjustment for the other covariates listed in Table 1 (including household income) did not substantially change the effect estimates. We evaluated the joint effect of mood and anxiety disorder history and pre-pregnancy overweight status. We classified women by the joint distribution of history of psychiatric disorder (no vs. yes) and pre-pregnancy overweight status (<25 vs. ≥25 kg/m^2^) resulting in the following categories: no psychiatric disorder and lean; history of psychiatric disorder and lean; no psychiatric disorder and overweight; and history of psychiatric disorder and overweight. This analytical approach allowed estimating ORs for sleep complaints among lean women with psychiatric disorder (i.e., to isolate the effect of psychiatric disorder, independent of overweight/obese status) and the ORs for sleep complaints among overweight women without psychiatric disorder (i.e. to isolate the effect of overweight status, independent of psychiatric disorder) when using lean women without psychiatric disorder as the referent group. The joint effect (or combined effect of both psychiatric disorder and overweight status) is determined by comparing those positive for both characteristics with the referent group.

**Table 1:** Characteristics of the study population: according to a medical history of mood/anxiety disorder, Seattle and Tacoma, Washington, USA, 2003-2006

	**Physician diagnosed mood/anxiety**	
	**Yes**	**No**
	**N=68**	**N=1,264**
**Characteristics**	**%**	**%**
Maternal Age (years)	34.2 ± 4.9^1^	33.3 ± 4.4^1^
Maternal Age (years)		
<25	1.5	2.9
25-34	50.0	57.5
35-39	35.3	32.2
≥40	13.2	7.4
Non-Hispanic white race/ethnicity	94.1	87.5
Annual household income (US$)*		
<30,000	2.9	1.9
30,000-69,999	27.9	13.1
≥ 70,000	66.2	80.4
Missing	2.9	4.7
Nulliparous	55.9	59.7
High school or less education	4.4	2.7
Unmarried	11.8	8.2
Pre-gestational chronic hypertensive	7.4	3.9
Family history of diabetes mellitus	11.8	14.6
Family history of hypertension	48.5	50.5
Employed during pregnancy	77.9	78.7
Smoked during pregnancy*	13.2	4.7
No prenatal vitamin	2.9	2.5
No exercise during pregnancy	7.4	7.3
Pre-pregnancy body mass index (kg/m^2^)	24.1 ± 4.9^1^	23.5 ± 4.6^1^
Pre-pregnancy body mass index (kg/m^2^)*		
Lean (<18.5)	4.4	4.5
Normal (18.5-24.9)	57.4	70.8
Overweight (25.0-29.9)	29.4	17.3
Obese (≥30.0)	8.8	7.4

^1^Mean ± standard deviation (SD).

^*^P-value <0.05 from Student t test for continuous variable or from Chi-square test for categorical variables.

All analyses were performed using Stata 9.0 statistical software (Stata, College Station, TX). All continuous variables are presented as mean ± standard deviation (SD). All reported confidence intervals were calculated at the 95% level. All reported p-values are two-tailed.

## Results

Approximately 5.1% of the cohort (n=68) reported having a medical diagnosis of depression or anxiety disorder prior to the interview. These included 55 women with a diagnosis of mood disorder, 10 women with a diagnosis of anxiety disorder and 3 women with comorbid mood and anxiety disorder. No participant reported having a diagnosis of bipolar disorder. Selective serotonin re-uptake inhibitors (SSRIs) were the most commonly used antidepressants (53 of 58), whereas benzodiazepines were the most commonly used anxiolytic medications (3 of 13). Women with a history of physician diagnosed mood or anxiety disorder were more likely to be in the lower annual household income bracket, to report smoking during pregnancy, and to be overweight or obese when compared with women who did not have a history of mood or anxiety disorders (Table 1). Other characteristics including marital status, race/ethnicity, physical activity and multivitamin use during pregnancy were similar for women with and without a history of mood or anxiety disorders. Descriptive statistics of maternal sleep complaint and stress variables are summarized in Table 2. Women with a history of mood or anxiety disorder were more likely to report short sleep duration (≤ 6 hours) during pregnancy, as compared with those with no such history. Similarly women with psychiatric disorders reported more frequent feelings of vital exhaustion and their perceived stress scores were higher than those reported by other women. The two groups were similar with respect to their Epworth sleepiness scores.

**Table 2:** Sleep complaints reported by pregnant women: with and without a medical history of mood/anxiety disorder, Seattle and Tacoma, Washington, USA, 2003-2006

	**Physician diagnosed mood/anxiety**		
	**Yes**	**No**	
	**N=68**	**N=1,264**	
**Sleep complaints**	**Mean ± SD or %**	**Mean ± SD or %**	**P-value**
Sleep hours during pregnancy (hours)	7.6 ± 1.7	7.8 ± 1.3	0.34^*^
≤6	23.5	13.7	0.08^**^
7-8	45.6	55.0	
≥9	29.4	30.8	
Missing	1.5	0.5	
Epworth sleepiness scale during pregnancy (score)	8.0 ± 3.4	7.6 ± 3.4	0.39^*^
Normal (0–9)	66.2	72.3	0.53^**^
Borderline (10–12)	22.0	18.8	
Abnormal (13–24)	11.8	8.9	
Vital exhaustion during pregnancy			
Never	27.9	52.7	<0.001^**^
Infrequently	29.4	23.7	
Frequently	17.7	9.8	
Very frequently	25.0	12.9	
Missing	0.0	0.9	
Perceived stress scale (score)	5.1 ± 2.5	3.8 ± 2.3	<0.001^*^
0-3	25.0	47.4	<0.001^**^
4-6	44.1	39.4	
7-12	30.9	10.7	
Missing	0.0	2.5	

^*^P-value from Student’s t test for continuous variable; ^**^P-value from Chi-square test.

Women with a history of mood or anxiety disorder were more likely to report sleeping ≤6 hours nightly during early pregnancy (OR=2.06; 95% CI 1.10-3.85) than those without the history. After adjusting for maternal age, race/ethnicity, smoking during pregnancy and pre-pregnancy body mass index, the association was attenuated but remained statistically significant (aOR=1.95; 95% CI 1.03-3.69). The adjusted odds of excessive daytime sleepiness (aOR=1.32; 95% CI 0.61-2.66), vital exhaustion (aOR=2.41; 95% CI 1.46-4.00) and elevated perceived stress during pregnancy (aOR=3.33; 95% CI 1.89-5.88) were elevated among women with mood or anxiety disorders compared with those without the disorders, though only the latter two conditions reached statistical significance (Table 3).

**Table 3:** Odds ratios (OR) and 95% confidence intervals (CI) of sleep complaints: according to medical history of mood/anxiety disorders, Seattle and Tacoma, Washington, USA, 2003-2006

	**Physician diagnosed mood/anxiety diagnosis**			
	**Yes**	**No**	**Unadjusted**	Adjusted^*^
	**N=68**	**N=1,264**		
**Sleep complaint**	**%**	**%**	**OR (95% CI)**	**OR (95% CI)**
Sleep duration during pregnancy				
Short (≤6 hours)	23.5	13.7	**2.06 (1.10-3.85)**	**1.95 (1.03-3.69)**
Normal (7–8 hours)	45.6	55.0	1.00 (Reference)	1.00 (Reference)
Long (≥9 hours)	29.4	30.8	1.15 (0.65-2.05)	1.13 (0.63-2.03)
Missing	1.5	0.5		
Excessive daytime sleepiness during pregnancy				
No	88.2	91.1	1.00 (Reference)	1.00 (Reference)
Yes	11.8	8.9	1.36 (0.63-2.91)	1.32 (0.61-2.86)
Vital exhaustion during pregnancy				
No	57.3	76.4	1.00 (Reference)	1.00 (Reference)
Yes	42.7	22.7	**2.50 (1.52-4.12)**	**2.41 (1.46-4.00)**
Missing	0.0	0.9		
Elevated perceived stress score (≥7) during pregnancy				
No	69.1	86.8	1.00 (Reference)	1.00 (Reference)
Yes	30.9	10.7	**3.63 (2.11-6.26)**	**3.33 (1.89-5.88)**
Missing	0.0	2.5		

^*^Adjusted for maternal age, race/ethnicity, smoking during pregnancy and pre-pregnancy body mass index.

We evaluated the joint effect of psychiatric disorder history and pre-pregnancy overweight status (Table 4) and noted that overweight women with a positive history of mood or anxiety disorder had the highest odds of short sleep duration during pregnancy, vital exhaustion and elevated perceived stress during pregnancy. Compared with lean women without psychiatric disorders, the multivariable-adjusted ORs among overweight/obese women reporting short sleep duration during early pregnancy was 2.88 (95% CI 1.14-7.32). Statistically significant associations were also observed for vital exhaustion (aOR=3.12; 95% CI 1.41-6.91) and elevated perceived stress (aOR=5.58; 95% CI 2.40-12.96) during pregnancy.

**Table 4:** Interaction of mood/anxiety and pre-pregnancy overweight status: adjusted odds ratios (AOR) and 95% confidence intervals (CI) of sleep complaints according to maternal history of mood/anxiety disorder and pre-pregnancy overweight status^**^, Seattle and Tacoma, Washington, USA, 2003-2006

	**No mood/anxiety**		**Yes mood/anxiety**		**No mood/anxiety**		**Yes mood/anxiety**	
	**No overweight**		**No overweight**		**Yes overweight**		**Yes overweight**	
	**(N=951)**		**(N=42)**		**(N=213)**		**(N=26)**	
**Sleep complaint**	**%**	**AOR (95% CI)**	**%**	**AOR (95% CI)**	**%**	**AOR (95% CI)**	**%**	**AOR (95% CI)**
Sleep duration during pregnancy								
Short (≤6 hours)	12.0	1.00 (Reference)	19.1	2.02 (0.86-4.74)	19.2	**1.73 (1.21-2.48)**	30.8	**2.88 (1.14-7.32)**
Long (≥9 hours)	31.3	1.00 (Reference)	33.3	1.29 (0.63-2.63)	29.1	1.02 (0.75-1.37)	23.1	0.89 (0.33-2.43)
Excessive daytime sleepiness during pregnancy	7.9	1.00 (Reference)	11.9	1.61 (0.61-4.25)	12.1	**1.58 (1.04-2.39)**	11.5	1.45 (0.42-5.02)
Vital exhaustion during pregnancy	21.0	1.00 (Reference)	40.5	**2.48 (1.31-4.69)**	27.8	**1.45 (1.08-1.94)**	46.2	**3.12 (1.41-6.91)**
Elevated perceived stress during pregnancy	9.9	1.00 (Reference)	23.8	**2.66 (1.24-5.70)**	13.1	1.30 (0.87-1.94)	42.3	**5.58 (2.40-12.96)**

^*^Adjusted for maternal age, race/ethnicity and smoking during pregnancy.

^**^None of the interactions reached statistical significance.

## Discussion

Approximately 5.1% of the cohort reported having a medical diagnosis of mood or anxiety disorder. Overall, women with a positive history of a psychiatric disorder were more likely than others to report short sleep durations, vital exhaustion and elevated perceived stress. The odds of these complaints were particularly elevated among overweight/obese women.

Substantial studies, generally conducted in men and non-pregnant women [11-13,15,17] or children [14,16] have examined one or more aspects of the mood or anxiety disorder-sleep comorbidity spectrum agree on our conclusion. Overall, our observations of higher odds of reported short sleep duration, excessive daytime fatigue, vital exhaustion and stress in pregnant women with a history of mood and anxiety disorder are consistent with previous studies. Our results are also largely consistent with reports from earlier studies that have documented increased prevalence of sleep disorders, stress or fatigue among pregnant women with mood or anxiety disorders [23-25,27,28]. A summary of findings from prior studies are presented in Additional file 1 (Table). For instance, our observation of positive associations of short sleep duration with mood or anxiety disorder is consistent with reports from Field et al. [24] who evaluated the effect of depression on self-report measures of sleep disturbance among 253 pregnant women at approximately 22 and 32 weeks gestation. The authors noted that those who were classified as depressed (using Structured Clinical Interview for DSM-IV (SCID)) were more likely to report sleep disturbances compared with non-depressed ones. Field et al. [23], later in a larger study among 911 pregnant women who were predominantly from low and medium socioeconomic status, replicated the same finding. In both studies the psychiatric diagnoses were made by research associates who used the SCID diagnostic instrument and who were supervised by a clinical psychologist. Okun et al. [25] in their secondary data analyses study, compared sleep disturbances in depressed (N=59) and non-depressed (N=181) pregnant women. Depression was ascertained using the SCID instrument at 20 weeks of gestation and sleep variables were extracted from the Structured Interview Guide for the Hamilton Depression Rating Scale with Atypical Depression Supplement (SIGH-ADS) at 20, 30, and 36 weeks gestation. The authors found that depressed women had more fragmented sleep at each assessment (p values<0.05). However, sleep deprivation, as reflected by percentage of total sleep duration <7 hrs (39.0% vs. 21.5%, p=0.008) and insomnia symptoms (55.9% vs. 30.4%, p=0.0004), was greater for depressed women only at 20 weeks gestation compared with non-depressed ones. Given that the sleep variables used in this study were imbedded within the SIGH-ADS depression screening scale, and thus were considered in making the psychiatric diagnosis, it is difficult to discern if observed correlations are independent of intrinsic measures of depression.

Overall, findings from the earlier studies provide some objective evidence on the relation between mood or anxiety disorders and sleep disturbances. However, methodological limitations including not controlling for confounding factors, small sample size, and lack of clinical diagnosis of mood/anxiety disorders did not allow one to draw firm conclusions to be made. Overcoming some of the noted methodological limitations of prior studies, our study adds to the body of evidence evaluating the impact of a clinically-diagnosed mood or anxiety disorders on sleep during early pregnancy. However, our results and those of others [23-25,27,28] are inconsistent with a report by Skouteris et al. [19]. Their results showed that depressive symptoms earlier in pregnancy did not impact sleep quality at a later stage in pregnancy.

Our observation of higher perceived stress score in women with mood/anxiety disorders are in agreement with previous literature [32]. Salacz and his colleagues found high Beck Depression Inventory (BDI) and State-Trait Anxiety Inventory (STAI) scores were associated with high levels of perceived stress (PSS score) in pregnancy women. A multiple regression analysis revealed that subjective feelings of stress explained over 50% of the variation in BDI and STAI scores [32].

Investigators speculated that common underlying pathophysiological neuroendocrine alterations involving the hypothalamus, serotonin and melatonin synthesis and secretion may, in part, explain consistent observations of increased risks of sleep disturbance in patients with mood and anxiety disorders. This hypothesis is supported by results from clinical [26,33-35] and functional neuroendocrine imaging studies [36,37]. Investigators have documented plasma melatonin circadian rhythm disturbances during pregnancy and postpartum in depressed women and women with personal or family histories of depression [26]. Moreover, investigators have reported lower plasma melatonin concentrations in patients with insomnia [33]. Melatonin is known to play a role in the biological regulation of circadian rhythms and sleep. Both animal and human studies demonstrated agomelatine (a melatonin analogue drug acting as melatonin agonist and 5-HT antagonist) to be an effective treatment for depression and bipolar disorder [34,35]. Serotonin secreted from the dorsal raphe nuclei is implicated in both the control of mood and anxiety disorder pathogenesis and sleep cycles [38]. The suprachiasmatic nucleus of the hypothalamus regulates the release of serotonin, supporting the thesis that the hypothalamus likely plays an important role in both the control of mood and sleep-regulating systems. Increased hypothalamic-pituitary-adrenal (HPA) activity, a robust pathophysiological biomarker associated with mood/anxiety disorders [39], is regarded as one important mechanism for observed associations between maternal psychiatric illness and perceived stress level [40].

Several limitations of our study merit discussion and consideration. First, the prevalence of mood/anxiety disorder of 5.1% in this sample is well below the national prevalence estimate reported by other investigators [21,22]. As a result, it is likely that a considerable number of women with psychiatric illnesses were misclassified as not having mood or anxiety disorder in our cohort. This under-ascertainment of psychiatric illnesses would generally serve to underestimate the true magnitude of associations detected in our study. Second, we did not collect information on onset of psychiatric symptoms, nor did we collection on the precise date of diagnosis. We were, however, able to distinguish women who had a recent diagnosis made prior to the study pregnancy or during the first 20 weeks of the index. We did not collect information of the effectiveness of the treatment. Third, maternal habitual sleep duration during pregnancy was obtained from self-report, and thus is likely susceptible to misclassification. Reported sleep duration is known to be only moderately correlated with wrist actigraph-measured sleep duration (*r*=0.47), and reports are generally longer by approximately 34 minutes for each hour of objectively measured sleep duration [41]. Future studies will require making objective assessments of maternal sleep duration. Fourth, we did not have information concerning participants’ shift-work or insomnia status and thus cannot attribute observed associations of short sleep duration with psychiatric disorders to occupational or medical conditions associated with short sleep duration. For instance, though related, short sleep duration and insomnia are different entities. Insomnia entails dissatisfaction with the quality of sleep and an inability to sleep given adequate opportunity. Insomnia can result in short sleep duration, but individuals with short sleep duration do not necessarily suffer from insomnia (i.e., participants may sleep less because they choose to do so or because they lack the opportunity to sleep). Future studies that allow for the comprehensive ascertainment of maternal sleep disorders (e.g., sleep disordered breathing, restless legs syndrome, insomnia, and circadian rhythm disorders) will be needed to more thoroughly assess the co-morbidity of psychiatric illnesses and sleep disorders among pregnant women. Fifth, although we adjusted for several potential confounders, we cannot exclude the possibility of residual confounding due to misclassification of adjusted variables (e.g., maternal pre-pregnancy body mass index) or confounding by other unmeasured variables (e.g., maternal exposure to the shift work during pregnancy). In consideration of evidence suggesting that adiposity may be associated with both psychiatric illnesses and sleep disorders [42], we report results from models which allow for assessing the independent and joint effects of psychiatric diagnoses and overweight status on each sleep complaint variable. The amount of sleep has declined by 1.5 hours over the past century, accompanied by an important increase in obesity [42]. Lastly, the generalizability of our study may also be limited as our cohort was primarily comprised of Non-Hispanic White and well-educated women. The concordance of our results with those from other studies that have included racially, ethnically and geographically diverse populations [23-25,27,28], however, serve to attenuate these concerns.

## Conclusions

We found increased odds of short sleep duration, vital exhaustion and perceived stress among pregnant women with mood or anxiety disorders compared with pregnant women without such disorders. These associations were particularly strong among overweight/obese women with psychiatric illnesses. Despite noted study limitations, our results are consistent with a larger body of work documenting associations between psychiatric disorders and sleep disturbances in men, non-pregnant women and children. Large well designed prospective cohort studies that allow for the comprehensive examination of comorbidity of the full spectrum of severity of psychiatric illnesses (e.g., isolated mood disorders, isolated generalized anxiety disorders, post-traumatic stress disorder, co-morbid mood and anxiety disorders) and the full spectrum of sleep disorders in pregnant women are warranted. Such studies should include objective assessments of sleep duration and disorders and should include comprehensive assessments of environmental, behavioral, and genetic risk factors of maternal antepartum and postpartum psychiatric health. Enhanced understanding of the epidemiology and shared pathophysiological mechanisms between psychiatric illnesses and sleep disturbances are expected to provide important information needed for enhancing the diagnosis and treatment of these disorders in pregnant women.

## Competing interest

The authors have no financial conflicts of interest.

## Authors’ contributions

CQ and MAW had full access to all the data in the study and take responsibility for the integrity of the data, the accuracy of the data analysis, and the decision to submit for publication. MAW conceived, designed and obtained funding for the study. CQ analyzed the data. CQ, BG, NF and MAW, drafted the manuscript. CQ, BG, NF and MAW interpreted the data, critically revised the draft for important intellectual content, and gave final approval of the manuscript to be published. All authors read and approved the final manuscript.

## Pre-publication history

The pre-publication history for this paper can be accessed here:

http://www.biomedcentral.com/1471-2393/12/104/prepub

## Supplementary Material

Additional file 1 **Supplementary Table.** Summaries of epidemiologic studies examining risk of sleep disorders among pregnant women with psychiatric disorders. Click here for file

## References

[B1] Chang JJ Pien GW Duntley SP Macones GA Sleep deprivation during pregnancy and maternal and fetal outcomes: is there a relationship? Sleep Med Rev 2010 14 107 114 19625199 10.1016/j.smrv.2009.05.001 PMC2824023

[B2] Dorheim SK Bondevik GT Eberhard-Gran M Bjorvatn B Sleep and depression in postpartum women: a population-based study Sleep 2009 32 847 855 19639747 10.1093/sleep/32.7.847 PMC2704916

[B3] Goyal D Gay CL Lee KA Patterns of sleep disruption and depressive symptoms in new mothers J Perinat Neonatal Nurs 2007 21 123 129 17505232 10.1097/01.JPN.0000270629.58746.96

[B4] Lee KA Gay CL Sleep in late pregnancy predicts length of labor and type of delivery Am J Obstet Gynecol 2004 191 2041 2046 15592289 10.1016/j.ajog.2004.05.086

[B5] Strange LB Parker KP Moore ML Strickland OL Bliwise DL Disturbed sleep and preterm birth: a potential relationship? Clin Exp Obstet Gynecol 2009 36 166 168 19860360

[B6] Williams MA Aurora SK Frederick IO Qiu C Gelaye B Cripe SM Sleep duration, vital exhaustion and perceived stress among pregnant migraineurs and non-migraineurs BMC Pregnancy Childbirth 2010 10 72 21047418 10.1186/1471-2393-10-72 PMC2987887

[B7] Lee KA Zaffke ME McEnany G Parity and sleep patterns during and after pregnancy Obstet Gynecol 2000 95 14 18 10636494 10.1016/s0029-7844(99)00486-x

[B8] Hedman C Pohjasvaara T Tolonen U Suhonen-Malm AS Myllyla VV Effects of pregnancy on mothers' sleep Sleep Med 2002 3 37 42 14592252 10.1016/s1389-9457(01)00130-7

[B9] Pien GW Schwab RJ Sleep disorders during pregnancy Sleep 2004 27 1405 1417 15586794 10.1093/sleep/27.7.1405

[B10] Dzaja A Arber S Hislop J Kerkhofs M Kopp C Pollmacher T Polo-Kantola P Skene DJ Stenuit P Tobler I Women's sleep in health and disease J Psychiatr Res 2005 39 55 76 15504424 10.1016/j.jpsychires.2004.05.008

[B11] Ohayon MM Interactions between sleep normative data and sociocultural characteristics in the elderly J Psychosom Res 2004 56 479 486 15172203 10.1016/j.psychores.2004.04.365

[B12] Agargun MY Kara H Solmaz M Sleep disturbances and suicidal behavior in patients with major depression J Clin Psychiatry 1997 58 249 251 9228889 10.4088/jcp.v58n0602

[B13] Breslau N Roth T Rosenthal L Andreski P Sleep disturbance and psychiatric disorders: a longitudinal epidemiological study of young adults Biol Psychiatry 1996 39 411 418 8679786 10.1016/0006-3223(95)00188-3

[B14] Chorney DB Detweiler MF Morris TL Kuhn BR The interplay of sleep disturbance, anxiety, and depression in children J Pediatr Psychol 2008 33 339 348 17991689 10.1093/jpepsy/jsm105

[B15] De Gennaro L Martina M Curcio G Ferrara M The relationship between alexithymia, depression, and sleep complaints Psychiatry Res 2004 128 253 258 15541782 10.1016/j.psychres.2004.05.023

[B16] Nevarez MD Rifas-Shiman SL Kleinman KP Gillman MW Taveras EM Associations of early life risk factors with infant sleep duration Acad Pediatr 2010 10 187 193 20347414 10.1016/j.acap.2010.01.007 PMC2866807

[B17] Selvi Y Aydin A Boysan M Atli A Agargun MY Besiroglu L Associations between chronotype, sleep quality, suicidality, and depressive symptoms in patients with major depression and healthy controls Chronobiol Int 2010 27 1813 1828 20969525 10.3109/07420528.2010.516380

[B18] Ford DE Cooper-Patrick L Sleep disturbances and mood disorders: an epidemiologic perspective Depress Anxiety 2001 14 3 6 11568977 10.1002/da.1041

[B19] Skouteris H Germano C Wertheim EH Paxton SJ Milgrom J Sleep quality and depression during pregnancy: a prospective study J Sleep Res 2008 17 217 220 18482110 10.1111/j.1365-2869.2008.00655.x

[B20] Bennett HA Einarson A Taddio A Koren G Einarson TR Prevalence of depression during pregnancy: systematic review Obstet Gynecol 2004 103 698 709 15051562 10.1097/01.AOG.0000116689.75396.5f

[B21] Kessler RC McGonagle KA Zhao S Nelson CB Hughes M Eshleman S Wittchen HU Kendler KS Lifetime and 12-month prevalence of DSM-III-R psychiatric disorders in the United States. Results from the National Comorbidity Survey Arch Gen Psychiatry 1994 51 8 19 8279933 10.1001/archpsyc.1994.03950010008002

[B22] Le Strat Y Dubertret C Le Foll B Prevalence and correlates of major depressive episode in pregnant and postpartum women in the United States J Affect Disord 2011 135 128 138 21802737 10.1016/j.jad.2011.07.004

[B23] Field T Diego M Hernandez-Reif M Figueiredo B Deeds O Ascencio A Schanberg S Kuhn C Comorbid depression and anxiety effects on pregnancy and neonatal outcome Infant Behav Dev 2010 33 23 29 19945170 10.1016/j.infbeh.2009.10.004 PMC2819543

[B24] Field T Diego M Hernandez-Reif M Figueiredo B Schanberg S Kuhn C Sleep disturbances in depressed pregnant women and their newborns Infant Behav Dev 2007 30 127 133 17292785 10.1016/j.infbeh.2006.08.002

[B25] Okun ML Kiewra K Luther JF Wisniewski SR Wisner KL Sleep disturbances in depressed and nondepressed pregnant women Depress Anxiety 2011 28 676 685 21608086 10.1002/da.20828 PMC3145808

[B26] Parry BL Meliska CJ Sorenson DL Lopez AM Martinez LF Nowakowski S Elliott JA Hauger RL Kripke DF Plasma melatonin circadian rhythm disturbances during pregnancy and postpartum in depressed women and women with personal or family histories of depression Am J Psychiatry 2008 165 1551 1558 18829869 10.1176/appi.ajp.2008.08050709 PMC3038788

[B27] Swanson LM Pickett SM Flynn H Armitage R Relationships among depression, anxiety, and insomnia symptoms in perinatal women seeking mental health treatment J Womens Health (Larchmt) 2011 20 553 558 21417746 10.1089/jwh.2010.2371

[B28] Jomeen J Martin CR Assessment and relationship of sleep quality to depression in early pregnancy Journal of Reproductive and Infant Psychology 2007 25 87 99

[B29] Kelman L Rains JC Headache and sleep: examination of sleep patterns and complaints in a large clinical sample of migraineurs Headache 2005 45 904 910 15985108 10.1111/j.1526-4610.2005.05159.x

[B30] Johns MW A new method for measuring daytime sleepiness: the Epworth sleepiness scale Sleep 1991 14 540 545 1798888 10.1093/sleep/14.6.540

[B31] Cohen S Kamarck T Mermelstein R A global measure of perceived stress J Health Soc Behav 1983 24 385 396 6668417

[B32] Salacz P Csukly G Haller J Valent S Association between subjective feelings of distress, plasma cortisol, anxiety, and depression in pregnant women Eur J Obstet Gynecol Reprod Biol 2012 [Epub ahead of print] 10.1016/j.ejogrb.2012.08.017 22948130

[B33] Attenburrow ME Dowling BA Sharpley AL Cowen PJ Case–control study of evening melatonin concentration in primary insomnia BMJ 1996 312 1263 1264 8634615 10.1136/bmj.312.7041.1263 PMC2351047

[B34] Gao K Calabrese JR Newer treatment studies for bipolar depression Bipolar Disord 2005 7 Suppl 5 13 23 16225556 10.1111/j.1399-5618.2005.00250.x

[B35] Jakovljevic M Agomelatine as chronopsychopharmaceutics restoring circadian rhythms and enhancing resilience to stress: a wishfull thinking or an innovative strategy for superior management of depression? Psychiatr Danub 2011 23 2 9 21448091

[B36] Ho AP Gillin JC Buchsbaum MS Wu JC Abel L Bunney WE Jr Brain glucose metabolism during non-rapid eye movement sleep in major depression. A positron emission tomography study Arch Gen Psychiatry 1996 53 645 652 8660131 10.1001/archpsyc.1996.01830070095014

[B37] Nofzinger EA Nichols TE Meltzer CC Price J Steppe DA Miewald JM Kupfer DJ Moore RY Changes in forebrain function from waking to REM sleep in depression: preliminary analyses of [18F]FDG PET studies Psychiatry Res 1999 91 59 78 10515462 10.1016/s0925-4927(99)00025-6

[B38] Passouant P Pituitary secretions and wake-sleep cycle Cephalalgia 1983 3 Suppl 1 42 53 6616608 10.1177/03331024830030S105

[B39] Lok A Mocking RJ Ruhe HG Visser I Koeter MW Assies J Bockting CL Olff M Schene AH Longitudinal hypothalamic-pituitary-adrenal axis trait and state effects in recurrent depression Psychoneuroendocrinology 2011 Nov 15. [Epub ahead of print] 10.1016/j.psyneuen.2011.10.005 22094110

[B40] Fernández-Guasti A Fiedler JL Herrera L Handa RJ Sex, stress, and mood disorders: at the intersection of adrenal and gonadal hormones Horm Metab Res 2012 44 607 618 22581646 10.1055/s-0032-1312592 PMC3584173

[B41] Lauderdale DS Knutson KL Yan LL Liu K Rathouz PJ Self-reported and measured sleep duration: how similar are they? Epidemiology 2008 19 838 845 18854708 10.1097/EDE.0b013e318187a7b0 PMC2785092

[B42] Garaulet M Ordovas JM Madrid JA The chronobiology, etiology and pathophysiology of obesity Int J Obes (Lond) 2010 34 1667 1683 20567242 10.1038/ijo.2010.118 PMC4428912

