# Supplementary material for: Short sleep duration, complaints of vital exhaustion and perceived stress are prevalent among pregnant women with mood and anxiety disorders
Source: BMC Pregnancy Childbirth. 2012 Oct 3;12:104. doi: 10.1186/1471-2393-12-104 (PMC3514205; doi:10.1186/1471-2393-12-104)
Supplement: Additional file 1 — Supplementary Table. Summaries of epidemiologic studies examining risk of sleep disorders among pregnant women with psychiatric disorders. [file 1471-2393-12-104-S1.doc]

**Suppl. Table**—Summaries of epidemiologic studies examining risk of sleep disorders among pregnant women with psychiatric disorders

| **Author**  **(Year)** | **Study Design** | **Sample**  **Size** | **Depression/Anxiety Assessment** | **Sleep Disorder Assessment** | **Study Findings** |
| --- | --- | --- | --- | --- | --- |
| **Okun *et al***  **(2011)** | Prospective cohort | 240 | Major depressive disorder using SCID | Sleep disorders questions extracted from the Structured Interview Guide for the Hamilton Depression Rating Scale with Atypical Depression Supplement (SIGH-ADS) | - Depressed women had more fragmented sleep at 20 and 30 weeks of gestation (P <0.05) - The frequency of insomnia symptoms was greater for depressed women only at 20 weeks gestation (e.g., Insomnia: 55.9% vs. 30.4%, P<0.001; sleep <7 hr: 39.0% vs. 21.5% , p=0.008) |
| **Swansoon *et al* (2011)** | Cross sectional | 114 | Depression using Edinburgh Postnatal Depression Scale (EPDS) and generalized anxiety (Penn State Worry Questionnaire [PSWQ]) | Insomnia using Insomnia Severity Index [ISI] | - After controlling for PSWQ, the partial correlation between EPDS and ISI was 0.15 (not significant) - After controlling for EPDS, the partial correlation between PSWQ and ISI was 0.20 (p<0.05) - For women having severe insomnia symptoms, the ORs for reporting significant symptom of depression and generalized anxiety symptoms are greater [OR=11.1 (95%CI 1.5-83.4), OR=3.6 (95%CI 1.3-9.8), correspondingly] |
| **Field *et al***  **(2010)** | Prospective cohort | 911 | Dysthymia, major depression, and anxiety disorder assessed using SCID | Sleep Disturbance Scale | - Women who experienced depression or anxiety were more likely to report sleep disturbances - The effect was more pronounced for those with comorbid conditions compared with the non-depressed group - Similar pattern was noted at 20 as well as 32 weeks |
| **Skouteris *et al***  **(2008)** | Prospective cohort | 273 | Beck Depression Inventory (BDI) | Pittsburgh Sleep Quality Index (PSQI) | - Sleep quality earlier in pregnancy predicted higher levels of depressive symptoms at later stage in pregnancy - Depressive symptoms earlier in pregnancy did not impact sleep quality at a late stage in pregnancy |
| **Parry *et al***  **(2008)** | Prospective cohort | 25 | Presence of depression assessed using SCID in conjunction with SIGH-ADS, BDI and EPDS scales | Plasma melatonin measured every 30 minutes during an overnight stay at the University of California general clinical research center | - Morning melatonin levels were significantly lower in pregnant depressives compared with non-depressed women - Pregnant women with a personal or family history of depression, regardless of current diagnosis, had significantly earlier melatonin synthesis and baseline offsets than those without such a history |

**Suppl. Table (Cont’d) Summaries of epidemiologic studies examining risk of sleep disorders among pregnant women with psychiatric disorders**

| **Author**  **(Year)** | **Study Design** | **Sample**  **Size** | **Depression/Anxiety Assessment** | **Sleep Disorder Assessment** | **Study Findings** |
| --- | --- | --- | --- | --- | --- |
| **Jomeen & Martin**  **(2007)** | Cross sectional | 148 | Presence of depression assessed using EPDS scale | Pittsburgh Sleep Quality Index (PSQI) | - Women classified as depressed were found to have significantly poorer sleep quality scores on the majority of PSQI sub-scales |
| **Field *et al***  **(2007)** | Prospective cohort | 253 | Dysthymia, major depression, and anxiety disorder assessed using SCID | Sleep Disturbance Scale | - During both second and third trimester, depressed women were more likely to have higher scores on sleep disturbances compared with non-depressed ones |
